# Connectomics underlying motor functional outcomes in the acute period following stroke

**DOI:** 10.3389/fnagi.2023.1131415

**Published:** 2023-02-15

**Authors:** Rong Bian, Ming Huo, Wan Liu, Negar Mansouri, Onur Tanglay, Isabella Young, Karol Osipowicz, Xiaorong Hu, Xia Zhang, Stephane Doyen, Michael E. Sughrue, Li Liu

**Affiliations:** ^1^Department of Rehabilitation, The First Affiliated Hospital of Nanjing Medical University, Nanjing, Jiangsu, China; ^2^University of Health and Rehabilitation Sciences, Qingdao, China; ^3^Department of Rehabilitation, The Affiliated Brain Hospital of Nanjing Medical University, Nanjing, Jiangsu, China; ^4^Omniscient Neurotechnology, Sydney, NSW, Australia; ^5^Xijia Medical Technology Company Limited, Shenzhen, China; ^6^International Joint Research Center on Precision Brain Medicine, Xidian Group Hospital, Xi'an, China

**Keywords:** stroke, machine learning, connectomic analysis, functional prediction, structural and functional connectivity, motor functional outcome, language networks, attention networks

## Abstract

**Objective:**

Stroke remains the number one cause of morbidity in many developing countries, and while effective neurorehabilitation strategies exist, it remains difficult to predict the individual trajectories of patients in the acute period, making personalized therapies difficult. Sophisticated and data-driven methods are necessary to identify markers of functional outcomes.

**Methods:**

Baseline anatomical T1 magnetic resonance imaging (MRI), resting-state functional MRI (rsfMRI), and diffusion weighted scans were obtained from 79 patients following stroke. Sixteen models were constructed to predict performance across six tests of motor impairment, spasticity, and activities of daily living, using either whole-brain structural or functional connectivity. Feature importance analysis was also performed to identify brain regions and networks associated with performance in each test.

**Results:**

The area under the receiver operating characteristic curve ranged from 0.650 to 0.868. Models utilizing functional connectivity tended to have better performance than those utilizing structural connectivity. The Dorsal and Ventral Attention Networks were among the top three features in several structural and functional models, while the Language and Accessory Language Networks were most commonly implicated in structural models.

**Conclusions:**

Our study highlights the potential of machine learning methods combined with connectivity analysis in predicting outcomes in neurorehabilitation and disentangling the neural correlates of functional impairments, though further longitudinal studies are necessary.

## 1. Introduction

Stroke is among the leading causes of long-term functional impairment worldwide (Karahan et al., 2014). Survivors often suffer residual motor, sensory, and cognitive deficits, all contributing to a deterioration in quality of life (Dobkin, 2005; Meyer et al., 2015). Beyond acute revascularization therapies, rehabilitation interventions remain paramount to aiding patients to regain function. However, one of the challenges in rehabilitation medicine is the heterogeneity in patient recovery trajectories after stroke. It remains difficult to predict whether or not patients will benefit from a given therapy, and it is clear that information beyond the observed deficits on assessment are necessary to prognosticate patients and prescribe individualized therapies which will improve quality of life. Machine learning methods may have a key role in traversing this gap and be a tool for prescribing precise treatments.

Several studies have already utilized machine learning algorithms to predict functional outcomes in the acute/subacute stroke period. Lin et al. compared the performance of several machine learning methods in predicting the Barthel Index (Lin W.-Y. et al., 2018). Other studies have assessed the ability of machine learning models in predicting cognitive, motor and sensory outcomes, many achieving high accuracy in classifying participants (Fang et al., 2021; Campagnini et al., 2022; Kim et al., 2022; Liao et al., 2022). Some of these studies have also examined the important features contributing to the model's classification. For example, Thakkar et al. found that the time since stroke, baseline functional independence, and baseline motor ability were the most predictive factors of motor function improvement in chronic stroke patients (Thakkar et al., 2020). It is unclear which set of features is most optimal to include in a predictive model, though it is likely that the input of these models must reflect the heterogeneity among patients. Moreover, it is unclear if there are any specific connectomic features that are predictive of outcome.

Magnetic resonance imaging (MRI)-based functional and structural connectivity may be potential candidates in prognosticating stroke outcomes, as they could provide personalized brain information at the individual level. Several studies have demonstrated an association between disruptions in structural and functional connectivity and stroke outcomes (Carter et al., 2010; Ding et al., 2014; Lin L. Y. et al., 2018; Puig et al., 2018; Lee et al., 2020). However, there has been limited attention on combining machine learning with connectivity analysis to predict functional outcomes; to-date, there have not been any exploratory analyses of whole brain connectomics predictive of post-stroke motor functional outcome in machine learning models. Here, we analyze functional connectivity (FC) and structural connectivity (SC) in a clinical sample, in order to (1) predict several stroke related motor functional outcomes, and (2) describe the connectomic correlates contributing to those predictions.

## 2. Methods

### 2.1. Patient cohort

A total of 79 first-time stroke patients with hemiplegia who were hospitalized in the Rehabilitation Medicine Department of The Affiliated Brain Hospital of Nanjing Medical University from April 2018 to December 2021 were prospectively included in this study. The study protocol was approved by the Ethics Committee of The Affiliated Brain Hospital of Nanjing Medical University, and written informed consent was obtained from all patients or their family members.

The inclusion criteria of the study included: (1) diagnosis of first-ever subcortical ischemic or hemorrhagic stroke by computed tomography or magnetic resonance; (2) patients were within 2–12 months after stroke; (3) patients only had unilateral limb hemiplegia (Modified Brunnstrom classification as grade I–IV); (4) patients did not have severe cognitive impairment; (5) patients did not have other acute diseases or serious complications; (6) right-handedness. The exclusion criteria were: (1) disturbance of consciousness, severe hearing and visual impairment; (2) significant pain in the affected side (Ten-point Visual Analog Scale > 4); (3) severe primary heart, lung, liver, kidney or hematopoietic system diseases; (4) after craniectomy or cranioplasty. (5) MRI contraindications.

### 2.2. Outcome assessment

The hospital records of the 79 patients were retrospectively reviewed for data collection. The following data on demographics and relevant past medical history were collected: age, sex, (hospitalization time, date of stroke onset and lesion location). Information on motor functional outcomes were also collected, using the following the assessment tools: Brunnstrom recovery stage (BRS) (Brunnstrom, 1966), modified Ashworth scale (MAS) (Bohannon and Smith, 1987), Barthel Index (Mahoney and Barthel, 1965), Fugl-Meyer Assessment (FMA) (Fugl-Meyer et al., 1975), functional ambulation category (FAC) (Holden et al., 1984), and Semans balance scale (Wang, 2018).

The functional classification of stroke patients was based on the BRS scale, which includes three parts: upper limb, lower limb and hand, with each part having six levels (0–6). The higher the level, the stronger the motor ability (Brunnstrom, 1966). The scale has good internal consistency in stroke patients. The MAS scale was used to evaluate spasticity in the biceps brachii and triceps surae muscles, which was graded out of six levels (0, 1, 1+, 2, 3, 4) (Bohannon and Smith, 1987). The higher the level, the more severe the spasm. The Barthel Index was used for the ability of daily living, with a total score of 100. The higher the score, the better the quality of life (Mahoney and Barthel, 1965). FMA was used to evaluate motor function, with a total score of 100, including 66 for upper limbs and 34 for lower limbs. The higher the score, the better the motor ability (Fugl-Meyer et al., 1975). FAC was used for assessing walking ability, and was divided into six levels—the higher the level, the better the walking function (Holden et al., 1984). The Semans balance scale was used to assess balance. It is an observational assessment method mainly used in the balance assessment of pediatric cerebral palsy and hemiplegic patients after stroke (Wang, 2018). It classifies balance into eight levels, with higher levels corresponding to better balance.

### 2.3. Imaging protocol

Image scanning was performed on the same day as functional assessment, on a 3.0-T MRI scanner (Siemens, Verio, Germany) in the Affiliated Brain Hospital of Nanjing Medical University. All patients lay supine with their head fixed by foam pads with a standard birdcage head coil to minimize head movement. Participants were instructed to remain as still as possible, open their eyes, remain awake, and not think of anything. High-resolution T1-weighted images were acquired by 3D magnetization-prepared rapid gradient-echo (MPRAGE) sequence [repetition time (TR) = 2,300 ms; echo time (TE) = 2.85 ms; flip angle (FA) = 9 degrees; matrix = 256 × 256; field of view (FOV) = 256 × 256 mm^2^; slice thickness/gap = 1/0.5 mm; 176 slices covered the whole brain] for image registration and functional localization. The imaging took ~260 s. The resting-state of functional MRI (rsfMRI) were subsequently collected in the same slice orientation with a gradient-recalled echo-planar imaging pulse sequence (TR = 2,000 ms; TE = 30 ms; FA = 90 degrees; matrix = 64 × 64, FOV = 240 × 240 mm^2^; thickness/gap = 4.0/0 mm; voxel size = 3.8 × 3.8 × 4 mm^3^; slice numbers = 30). A total of 251 volumes were obtained in this acquisition sequence and each functional resting-state session lasted ~500 s.

Diffusion-weighted imaging was performed with the SE-EPI sequence scanning. Using iPAT technology, axial scanning, TR = 11,000 ms, TE = 90 ms, slice number = 67, slice thickness = 2 mm, slice spacing 0 mm, FOV = 256 mm × 256 mm, matrix: 128 × 128, voxel size = 2 × 2 × 2 mm^3^, diffusion weighted scan b = 1,000 s/mm^2^ in 30 gradient directions, and another non-diffusion weighted image with b = 0. Excitation times (NEX) = 2. Scan time = 11 min and 57 s.

### 2.4. Diffusion weighted imaging pre-processing

Diffusion weighted imaging (DWI) was processed using the Infinitome software (Omniscient Neurotechnology, 2020). Infinitome relies on standard processing steps using Python (Garyfallidis et al., 2014). The diffusion image was resliced to obtain isotropic voxels. A rigid body alignment was used for motion correction, and slices with excess motion, defined as DVARS >2 sigma from the mean slice were eliminated. The T1 image was then skull stripped using a convolution neural net, described elsewhere (Isensee et al., 2019). This was then inverted and aligned to the DWI image using a rigid alignment, which was then used as a mask to skull strip the DWI image. A diffeomorphic warping method was then used to correct for gradient distortion to similarize the DWI and T1 images, and eddy current correction was performed. Finally, the fiber response function was estimated and constrained spherical deconvolution was used calculate the diffusion tensors. Deterministic tractography was then performed, with random seeding, manifesting in around 300,000 streamlines per brain (Doyen et al., 2022).

### 2.5. Structural-connectivity based parcellation

A parcellation scheme is necessary for measuring FC changes at an anatomical level. Most available atlasing methods to parcellate the brain into functional regions however are derived from healthy subjects. These atlases also are based on a group average of these healthy cohorts, and are therefore unable to account for individual morphological differences such as gyral variation. This is especially problematic when applying these atlases to brains altered by pathology, such as in stroke. In order to account for any morphological deficits caused by stroke, we utilized a machine learning based method to individually parcellate each brain based on the Human Connectome Project Multimodal Parcellation (HCP-MMP1) atlas (Glasser et al., 2016), creating a subject-specific atlas. While we have described this method in detail elsewhere (Doyen et al., 2022), briefly, a machine learning model was trained using DWI data from 178 healthy controls from the Schiz Connect database, which were pre-processed as above. The model consequently learnt the structural connectivity pattern between each voxel. The unalted HCP-MMP1 was then warped to each brain in the current study's cohort, and the machine learning model was applied to each brain to re-appoint voxels to their most likely parcellation based on structural connectivity features. This method manifested in “reparcellation” of voxels, creating a subject-specific version of the HCP-MMP1 atlas with 180 cortical regions, 9 subcortical regions, and the brainstem.

Each brain region was then automatically mapped to a known large-scale brain network by the Infinitome software, which itself relies upon previous meta-analyses mapping each large-scale brain network. The network template described by Yeo et al. (2011) was utilized, including the core networks: the Central Executive Network (CEN), Default Mode Network (DMN), Dorsal Attention Network (DAN), Limbic Network (LN), Salience Network (SN), Sensorimotor Network (SMN), and the Visual Network (VN), along with several networks which are either part of the extended versions of the core networks, or additional networks described in the literature, including the Accessory Language and Language Networks (part of the extended DMN), Auditory System (part of the SMN), Multiple demand network, and Ventral Attention Network (VAN).

### 2.6. Resting-state fMRI pre-processing steps

rsfMRI images were pre-processed prior to analysis according to standard pre-processing steps. A rigid body alignment was used to perform motion correction on the T1 and blood-oxygen-level-dependent (BOLD) images. Slices with excess movement (defined as DVARS > 2 sigma from the mean slice) were eliminated. A CNN was used to skull strip the T1 image (Isensee et al., 2019). In order to then skull strip the rsfMRI image, the T1 image was inverted and aligned to the BOLD image using a rigid alignment, and then used as a mask. Slice timing correction, global intensity normalization, and gradient distortion correction were performed, the latter using a diffeomorphic warping method to register the rsfMRI and T1 images. The CompCor methodwas used to calculate high variance confounds (Behzadi et al., 2007), which were regressed out of the rsfMRI image along with the motion confounds. The linear and quadratic signals were detrended (note this method does not perform global signal regression). A 4 mm FWHM Gaussian kernel was used to perform spatial smoothing. The subject-specific brain atlas created in the previous steps were then registered to the T1 image, and the regions were aligned with the regions in each subject's scan. A visual check was then performed by two neuroanatomists, independently, to ensure the methodology accounted for the morphological changes caused by the lesion. The atlas was therefore ideally positioned to extract a BOLD time series, averaged over all the voxels within a region, from 379 brain regions (180 cortical regions from two hemispheres, along with 19 subcortical structures). A Pearson correlation coefficient was then calculated between the BOLD signals of each unique pair of regions, yielding. Thus, it is ideally positioned for extracting a BOLD time series, averaged over all voxels within a region, from all 379 regions (180 parcels from two hemispheres, plus 19 subcortical structures). The Pearson correlation coefficient is calculated between the BOLD signals of each unique area pair (self to self-inclusive), which yields 71,631 unique correlations.

### 2.7. Machine learning classification and feature extraction

Machine learning was used to model the test performance of each participant based on the pairwise functional correlation or structural connectivity between the 379 regions of each individual's brain atlas. XGBoost Classifier (Chen and Guestrin, 2016), a boosted trees approach was used to fit each model. All models included age and sex as nuisance predictors. A total of 16 models were trained—one utilizing structural connectivity (SC), and another functional connectivity (FC)—to predict performance on the BRS on upper extremity, FMA on upper and lower extremities, MAS on upper and lower limbs, Barthel Index, FAC, and Semans balance scale. The scores for each of these tests were binarized for the models to classify subjects into either category, as detailed in Table 1.

**Table 1:** Cutoffs for each functional outcome scale utilized in the machine learning models.

**Functional outcome scale**	**Scoring cutoffs**	
Brunnstrom Stage Recovery (BRS)—upper extremity	Stages 1–3	Worse
	Stages 4–6	Better
Fugl-Meyer Assessment (FMA)—lower extremity	< 21	Worse capacity
	≥21	Better capacity
Fugl-Meyer Assessment (FMA)—upper extremity	≤ 31	Worse capacity
	≥32	Better capacity
Modified Ashworth Scale (MAS)—upper extremity	0	No muscle tone
	>0	Some muscle tone
Modified Ashworth Scale (MAS)—lower extremity	0	No muscle tone
	>0	Some muscle tone
Barthel index	≤ 40	Very or totally dependent
	41–100	Partially to fully independent
Semans balance	0–1	Severe
	>1	Moderate to none
Functional ambulation category	0	Non-ambulatory
	1–5	Ambulatory

Five-fold cross-validation was used for each model, with different training and test data splits to ensure the hyperparameters were not being optimized just for the given training and test data splits. The models were evaluated with the mean area under the receiver operating characteristic curve (AUC-ROC) ± standard deviation. Given the small sample size, several strategies were utilized to minimize the impact of class imbalances on the performance of each model. Five-fold cross-validation ensured that each fold had an equal ratio of both classes; a stopping criterion was applied to stop training if the model performance did not improve over consecutive iterations of hyperparameter tuning—an attempt to prevent overfitting; and, feature importance analysis only relied upon the correctly predicted cases, therefore being independent of whether one class was predicted slightly better than the other. These strategies aimed to approximate a balanced class AUC even in cases of class imbalance. Our predominant aim in each model was also feature importance analysis, with a focus on identifying what resulted in the classifications. Consequently, while AUC is inherently affected by class imbalances, the guardrails applied, along with the significantly higher than chance performance of each model counteract any AUC bias, and make it largely irrelevant to the aim of our analysis.

The feature importance analysis for each model was visualized using a SHAP plot of the top 20 features, either functional or structural connectivity between two regions of the HCP-MMP1 atlas, contributing to the model. The SHAP method, based on cooperative game theory, calculates Shapley values for each feature, which estimate the marginal contribution of each feature to the outcome of models relying on every possible permutation of features (Lundberg and Lee, 2017). Each SHAP plot shows a list of features in descending order of importance, quantifying the impact on the model along the x-axis, with the color of each point representing a single observation, indicating whether a high (red), or low (blue) value of that feature is associated with the model. A network-based summary plot is also produced, aggregating the contribution of each parcel by their network affiliation. This gives an indication of which brain networks are most influential in each model's classification.

## 3. Results

### 3.1. Subject demographics

Subject demographics are detailed in Table 2. The median age (±IQR) of our sample was 62 ± 18. There were 22 females (27.8%) and 57 males (72.2%). The median time (±IQR) from initial stroke to scanning was 35 days (33). There was a small difference in the stroke side, with 55.7% of patients having a left-sided stroke; however, speech deficits were only seen in 21.5% of patients, and most patients had a subcortical stroke. Motor deficits were present in 96.2% of patients.

**Table 2:** Subject demographics.

**Demographics**	***N* = 79**
Age *years (median ± IQR)*	62.0 ± 18.0
Sex F/M *n (%)*	22 (27.8)/57 (72.2)
Years of education *(median ± IQR)*	9.0 ± 6.0
Hand dominance L/R *n (%)*	79 (100)/0 (0)
Time from first admission to fMRI *days (median ± IQR)*	35 (33)
Stroke side^*^ L/R *n (%)*	44 (55.7)/34 (43.0)
**Stroke location** ***n (%)***	
Cortical	12 (15.2)
Subcortical	52 (65.8)
Both	15 (19.0)
**Deficits on admission** ***n (%)***	
Motor	76 (96.2)
Cognition	21 (26.6)
Speech	17 (21.5)
Dysarthria	21 (26.6)
Dysphagia	25 (31.6)
Ataxia	2 (2.5)
**Brunnstrom stage of recovery** ***(median** ±**IQR)***	
Upper limb	3 (3)
Lower limb	4 (3)
**Fugl-Meyer assessment** ***(median** ±**IQR)***	
Total motor	39 (53.5)
Upper limb motor	20 (37.5)
Lower limb motor	18 (22)
**Modified Ashworth scale** ***(median** ±**IQR)***	
Upper limb	0 (1)
Lower limb	0 (0.25)
Barthel index *(median ± IQR)*	50 (42.5)
Semans balance scale *(median ± IQR)*	3 (2)

* One subject had an infarct of the corpus callosum.

### 3.2. Models classifying tests of limb function and spasticity

Machine learning was applied to classify subjects into binarized categories as outlined in Table 1. For each test, two models were constructed to separately evaluate functional and structural connectivity. Both models included age and sex as covariates.

#### 3.2.1. Brunnstrom stage of recovery (BRS)—Upper extremity

**Figure 1:**
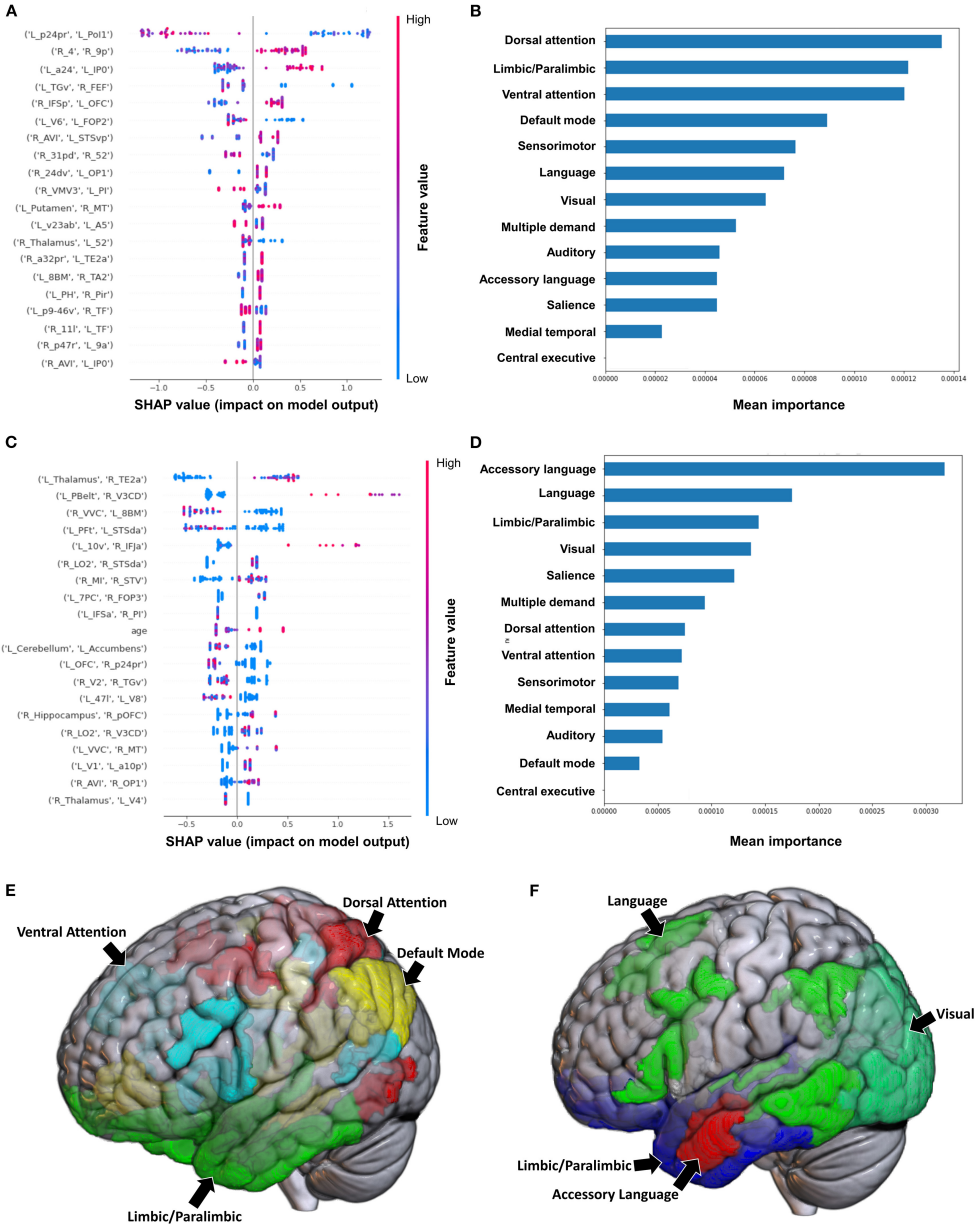
Feature importance analysis of the models classifying the Brunnstrom stage of recovery—upper extremity based on functional connectivity at the **(A)** individual brain region level, and **(B)** the network level; and based on structural connectivity at the **(C)** the individual brain region level, and **(D)** the network level. The networks contributing most to the models' classifications, based on **(E)** functional connectivity, and **(F)** structural connectivity, have also been visualized on brain models.

The model utilizing subjects' FC to classify the upper limb BRS achieved a mean (±SD) AUC of 0.81 ± 0.12. Utilizing the SHAP method for feature extraction revealed that at the individual brain region level, a low FC between the left area posterior 24 prime (p24pr) and left area posterior insular 1 (PoI1), a high FC between the right primary motor cortex (area 4) and right area 9 posterior (9p), and a high FC between left area anterior 24 (a24) and left area intraparietal 0 (IP0) contributed most to the model's classification (Figure 1A). The rest of the features did not demonstrate as significant an impact on the output. At the network level, the DAN, limbic/paralimbic system, and VAN had the largest contribution to the model's output, followed by the DMN (Figures 1B, E).

The model employing SC to classify the upper limb BRS achieved a mean (±SD) AUC of 0.66 ± 0.13. At the individual brain region level, the structural connectivity between the left Thalamus and right area TE2 anterior (TE2a) (though the direction of this connectivity was unclear), and a high connectivity between left parabelt complex (Pbelt) and right V3CD were the top two features (Figure 1C). However, there was significant variability and overlap among the feature contribution values, and this model therefore requires caution while interpreting. At the network level, the accessory language network demonstrated the greatest contribution to the model's classification (Figures 1D, F).

#### 3.2.2. Fugl-Meyer Assessment

**Figure 2:**
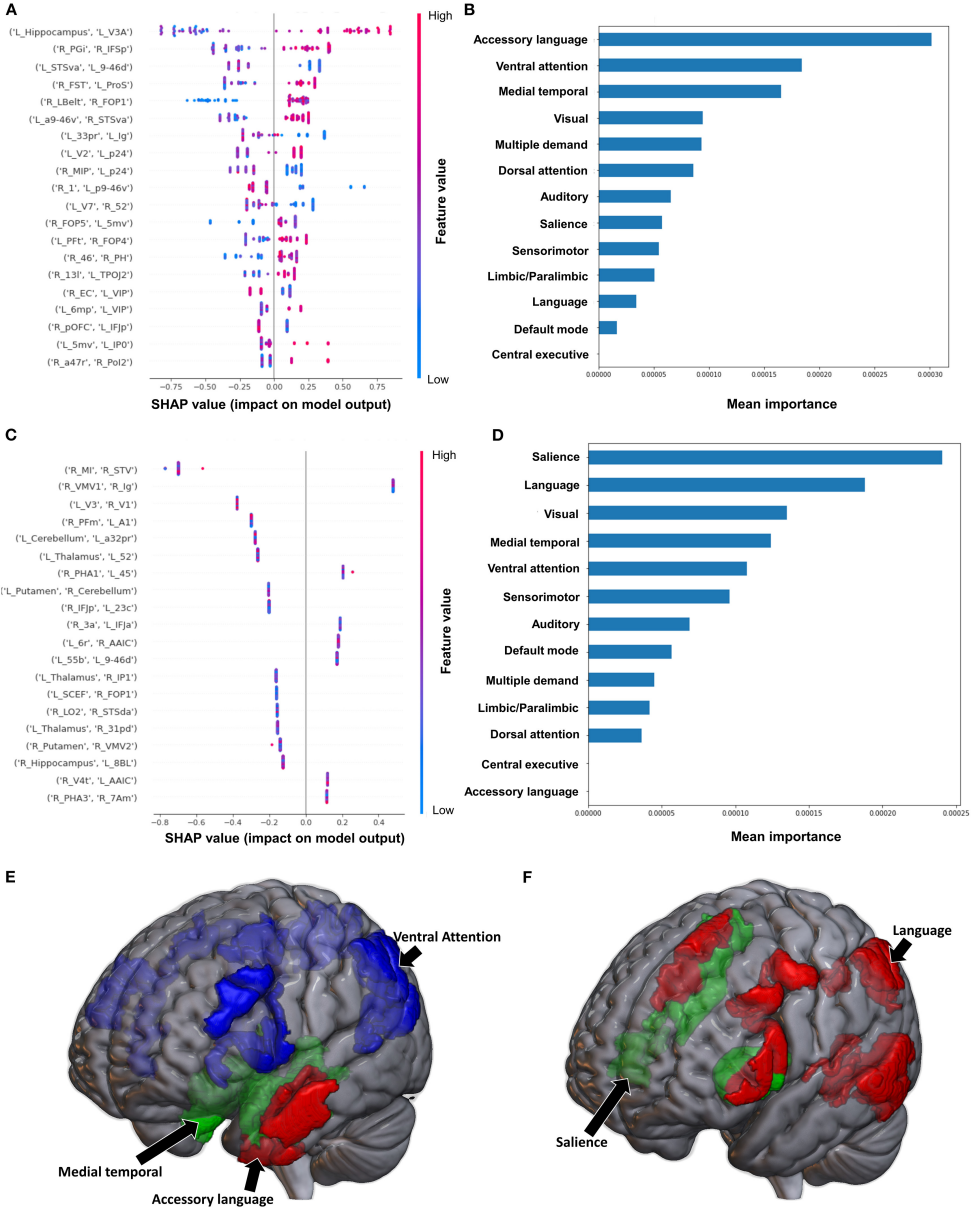
Feature Importance Analysis of the models classifying the Fugl Meyer assessment—lower extremity based on functional connectivity at the **(A)** individual brain region level, and **(B)** the network level; and based on structural connectivity at the **(C)** the individual brain region level, and **(D)** the network level. The networks contributing most to the models' classifications, based on **(E)** functional connectivity, and **(F)** structural connectivity, have also been visualized on brain models.

The model classifying the FMA lower extremity score using FC achieved a mean AUC of 0.797 ± 0.052. A high FC between the left hippocampus and left V3A had the greatest impact on the model's output, while the rest of the features demonstrated a mixed pattern of FC, with great overlap (Figure 2A). At the network level, the accessory language network demonstrated the greatest contribution, followed by the VAN and medial temporal regions (Figures 2B, E). When utilizing SC, the model achieved a mean AUC of 0.650 ± 0.117. The model performance and the low level of variance among the subjects limited further interpretation of this model, especially at the individual region level (Figure 2C), though the SN and language network demonstrated the greatest contribution at the network level (Figures 2D, F).

**Figure 3:**
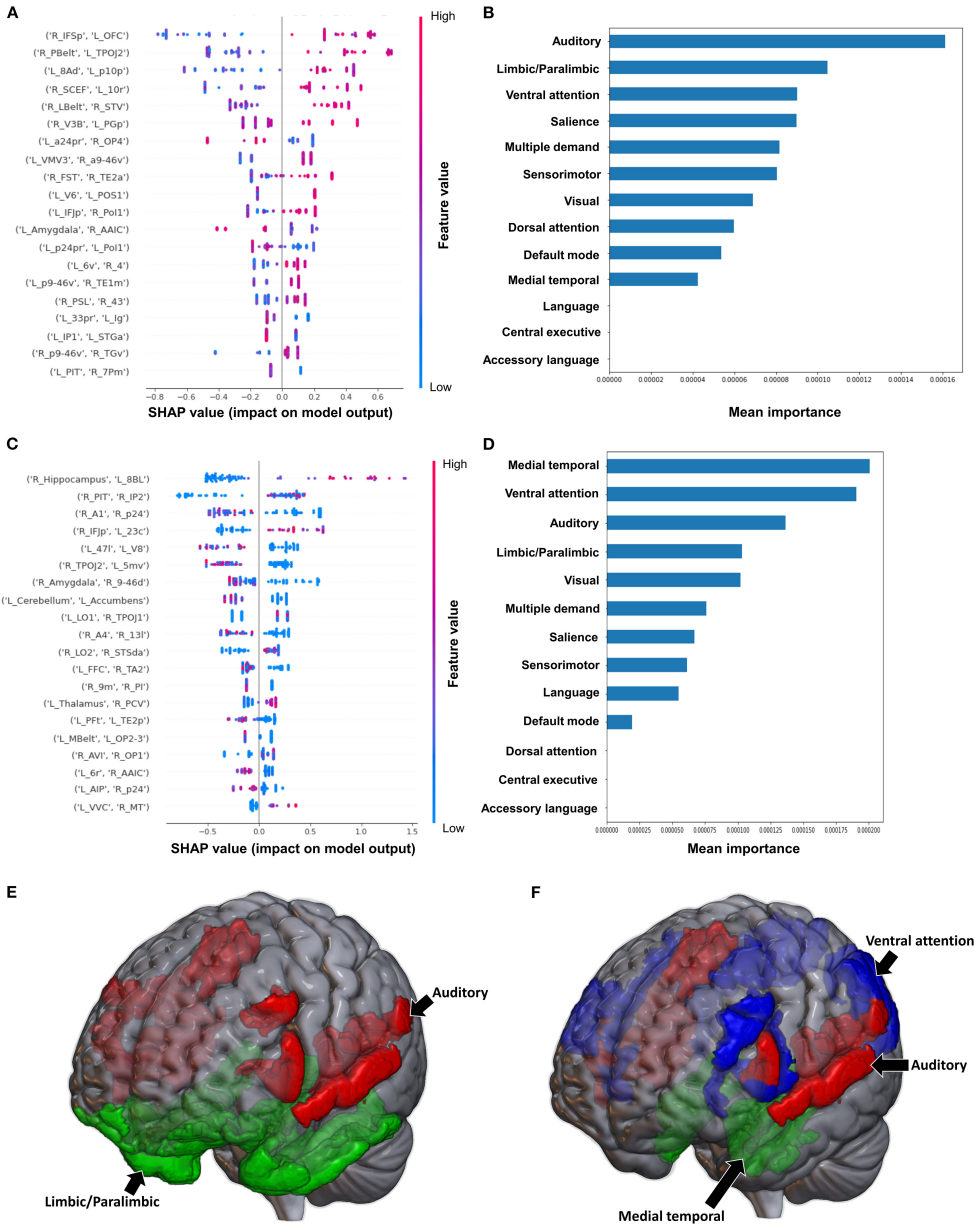
Feature Importance Analysis of the models classifying the Fugl Meyer assessment—upper extremity based on functional connectivity at the **(A)** individual brain region level, and **(B)** the network level; and based on structural connectivity at the **(C)** the individual brain region level, and **(D)** the network level. The networks contributing most to the models' classifications, based on **(E)** functional connectivity, and **(F)** structural connectivity, have also been visualized on brain models.

Classification of the upper extremity FMA scores using FC yielded a mean AUC of 0.677 ± 0.080. At the individual region level, a high FC between the right IFSp and left orbital frontal complex (OFC), right PBelt and left temporoparietooccipital junction (TPOJ2), left dorsal area 8A (8Ad) and left area posterior 10p (p10p), right supplementary and cingulate eye field (SCEF) and left rostral area 10 (10r), right lateral belt complex (LBelt) and right superior temporal visual area (STV) were the top five features contributing to the model's output (Figure 3A). At the network level, the auditory system, followed by the limbic/paralimbic system had the greatest contribution (Figures 3B, E). When utilizing structural connectivity to classify upper extremity FMA scores, the model demonstrated a mean AUC of 0.788 ± 0.045. A high connectivity between the right hippocampus and left area 8B lateral (8BL) was the top feature contributing to the model's output, while the rest of the features generally were associated with low connectivity, though there was too much overlap to highlight a single feature and these features did not show as a significant contribution to the model (Figure 3C). At the network level, the medial temporal regions and VAN, followed by the auditory system demonstrated the greatest contribution to the output (Figures 3D, F).

#### 3.2.3. Modified Ashworth scale

**Figure 4:**
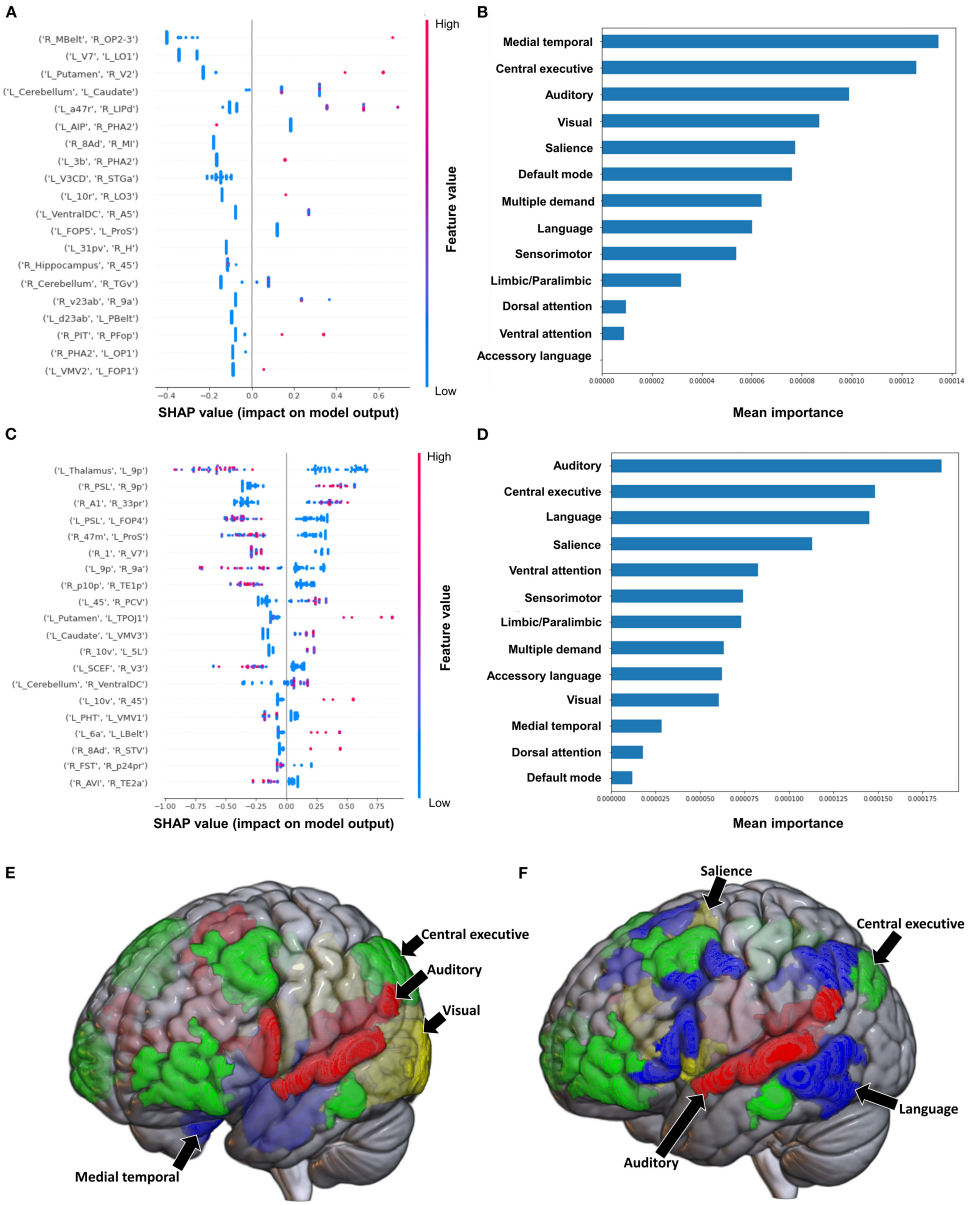
Feature Importance Analysis of the models classifying the modified Ashworth scale—lower extremity based on functional connectivity at the **(A)** individual brain region level, and **(B)** the network level; and based on structural connectivity at the **(C)** the individual brain region level, and **(D)** the network level. The networks contributing most to the models' classifications, based on **(E)** functional connectivity, and **(F)** structural connectivity, have also been visualized on brain models.

The model classifying the MAS lower extremity score into no change in muscle tone and some change in muscle tone achieved a mean AUC of 0.753 ± 0.140. Due to the relative class imbalance of this model, the SHAP output was difficult to interpret at the individual brain region level, and while a high FC between the right medial belt complex (Mbelt) and right posterior operculum 2-3 (OP2-3) had the highest contribution to the output, it should be interpreted with caution (Figure 4A). At the network level however, the medial temporal regions and CEN, followed by the auditory system and VN demonstrated the greatest contribution to the model's output (Figures 4B, E). When utilizing SC to classify MAS lower extremity scores, the model achieved a mean AUC of 0.802 ± 0.108. A low connectivity between the left thalamus and left area 9 posterior (9p) had the greatest contribution to the model's output, and the top 10 features generally pointed to low connectivity (Figure 4C). There was however significant overlap among the features, making it difficult to ascertain the relative importance of features. At the network level, the auditory system and CEN were once again among the top four networks contributing to the model's output, and were followed by the language system and SN (Figures 4D, F).

**Figure 5:**
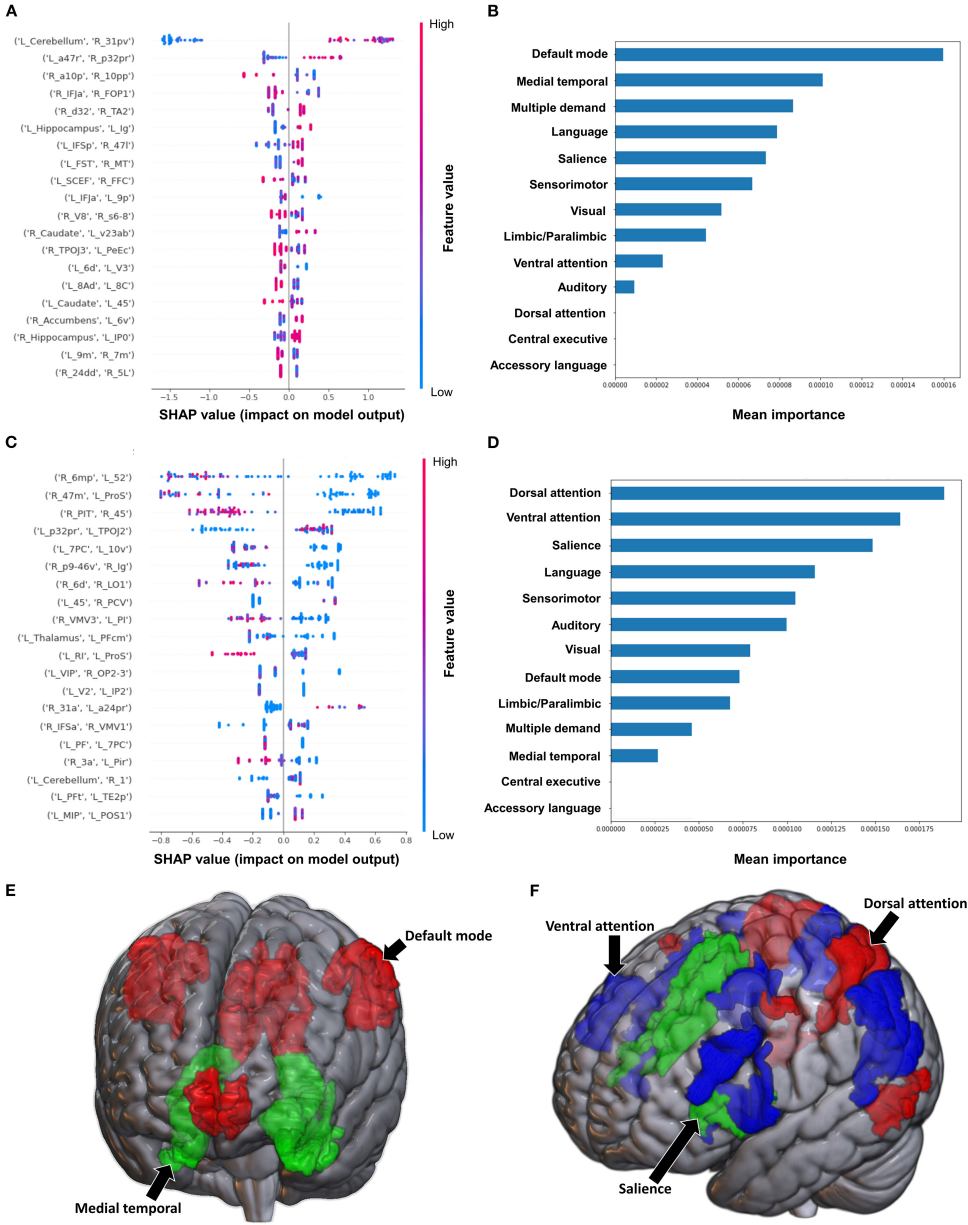
Feature Importance Analysis of the models classifying the modified Ashworth scale—upper extremity based on functional connectivity at the **(A)** individual brain region level, and **(B)** the network level; and based on structural connectivity at the **(C)** the individual brain region level, and **(D)** the network level. The networks contributing most to the models' classifications, based on **(E)** functional connectivity, and **(F)** structural connectivity, have also been visualized on brain models.

Classification of the upper extremity scores using FC yielded a mean AUC of 0.815 ± 0.080. At the individual region level, a high FC between the left cerebellum and right area 31p ventral (31pv) had the greatest contribution to the model's output, followed by a high FC between left area anterior 47r (a47r) and right area p32 prime (p32pr) (Figure 5A); while at the network level, the DMN contributed most to the output, followed by medial temporal regions (Figures 5B, E). When utilizing structural connectivity to classify upper extremity scores, the model demonstrated a mean AUC of 0.737 ± 0.150. A low SC between the right area 6mp and left area 52, right area 47m and left prostriate area (ProS), and right posterior inferotemporal complex (PIT) and right area 45 were the top three features contributing to the model's classification (Figure 5C). At the network level, the DAN, VAN, and SN demonstrated the greatest contribution (Figures 5D, F).

### 3.3. Models classifying tests of general functional performance activities of daily living

#### 3.3.1. Barthel Index

**Figure 6:**
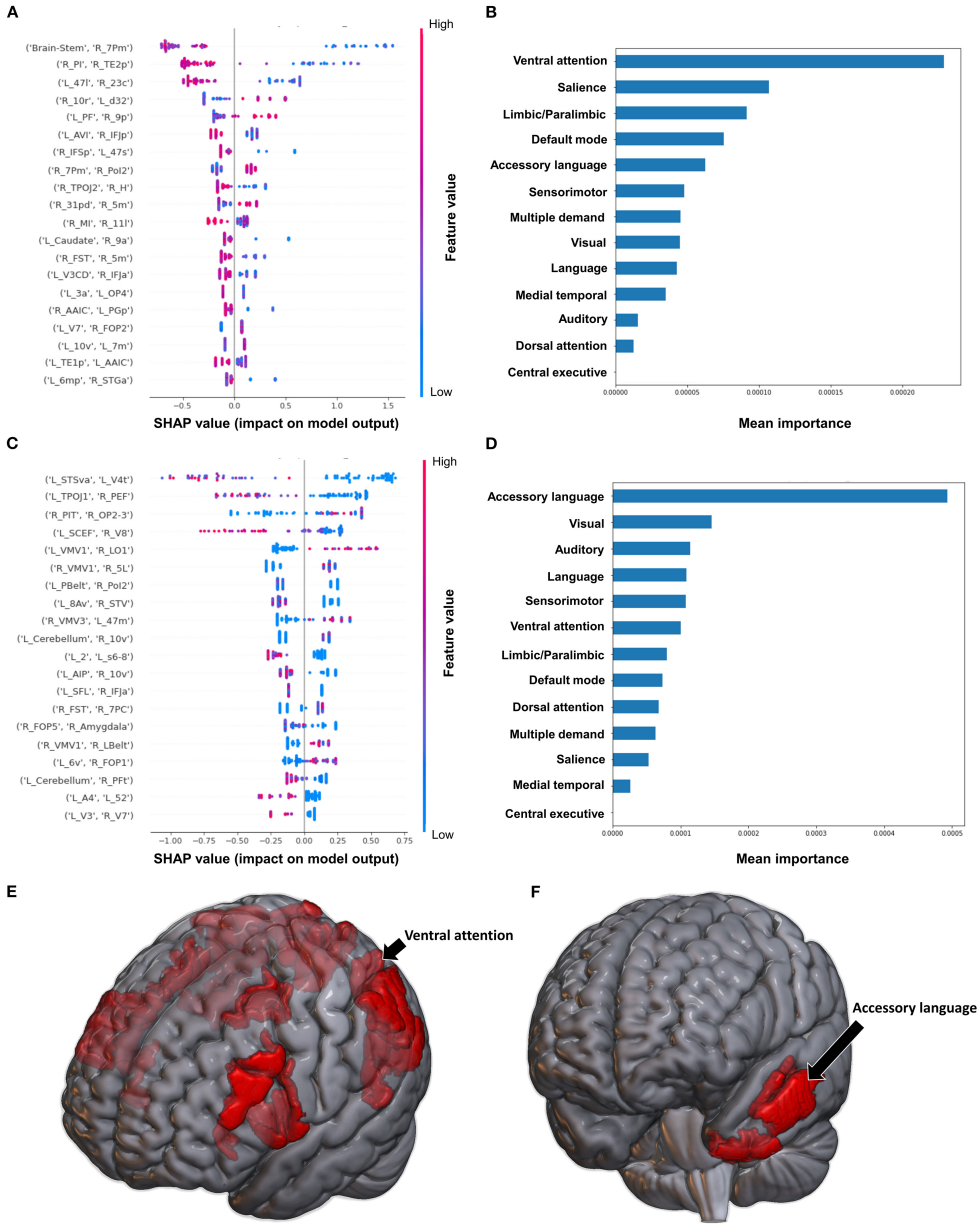
Feature Importance Analysis of the models classifying the Barthel index based on functional connectivity at the **(A)** individual brain region level, and **(B)** the network level; and based on structural connectivity at the **(C)** the individual brain region level, and **(D)** the network level. The networks contributing most to the models' classifications, based on **(E)** functional connectivity, and **(F)** structural connectivity, have also been visualized on brain models.

When using FC to classify subjects based on the Barthel Index, the model achieved a mean AUC of 0.808 ± 0.094. A low FC between the brainstem and right medial area 7P (7Pm), and right para-insular area (PI) and right area TE2 posterior (TE2p) were the top 2 features contributing to the model's classification, while the rest of the features demonstrated great overlap in contribution (Figure 6A). The VAN had the greatest contribution at the network level (Figures 6B, E).

The model utilizing SC achieved a mean AUC of 0.742 ± 0.175. Low connectivity between the left area superior temporal sulcus ventral-anterior (STSva) and left area V4t, and left area temporoparietooccipital junction 1 (TPOJ1) and right premotor eye field (PEF) had the greatest impact on the model's output, though there was once again significant overlap in contribution values (Figure 6C). At the network level, the accessory language network had the greatest contribution to the model's classification (Figures 6D, F).

#### 3.3.2. Semans balance scale

**Figure 7:**
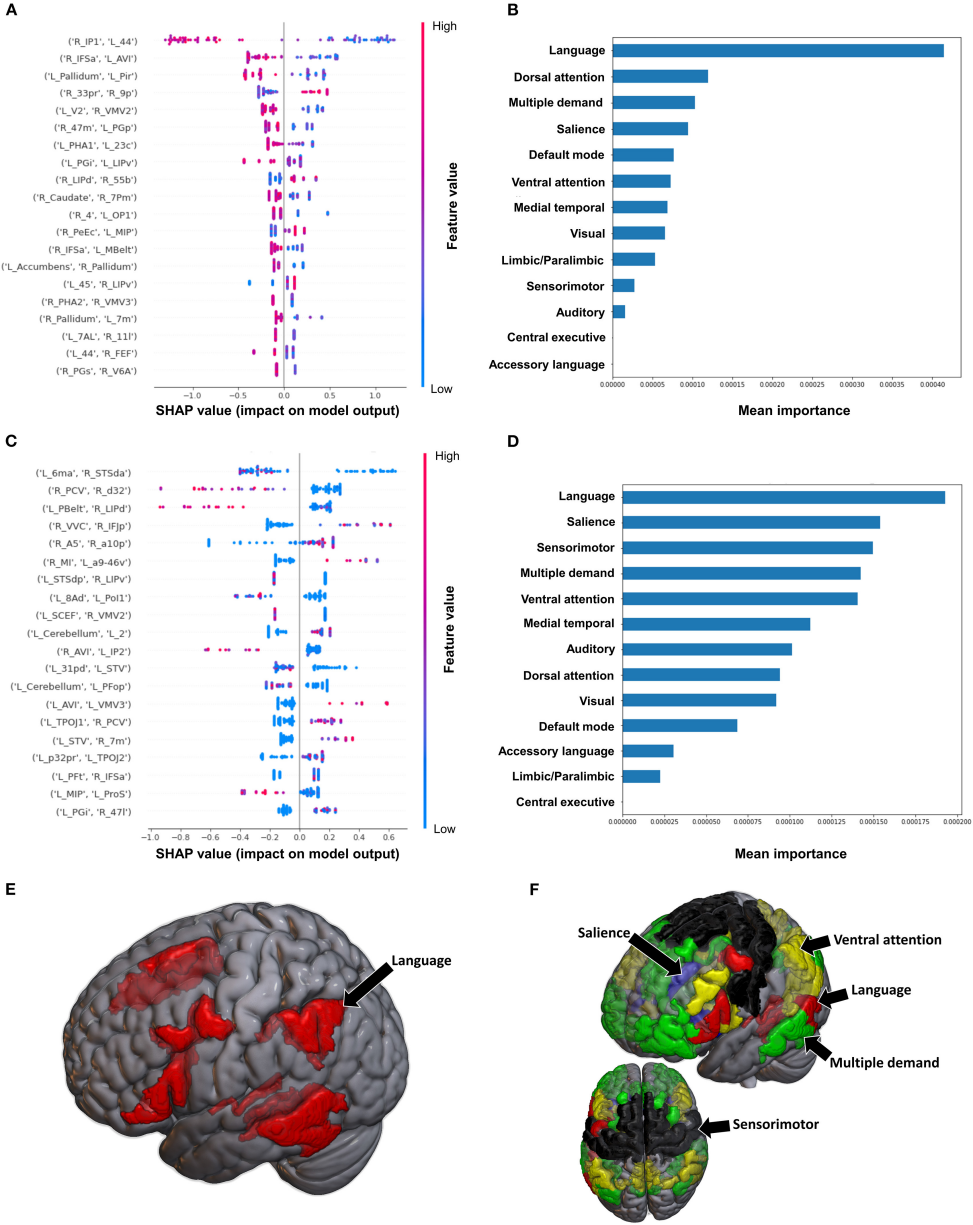
Feature Importance Analysis of the models classifying the Semans balance scale based on functional connectivity at the **(A)** individual brain region level, and **(B)** the network level; and based on structural connectivity at the **(C)** the individual brain region level, and **(D)** the network level. The networks contributing most to the models' classifications, based on **(E)** functional connectivity, and **(F)** structural connectivity, have also been visualized on brain models.

The model utilizing FC to classify balance performance yielded a mean AUC of 0.868 ± 0.155. A low FC between the right area intraparietal 1 (IP1) and left area 44 had the greatest contribution to the model's output (Figure 7A), while at the network level, the language system was the top network (Figures 7B, E). The model utilizing structural connectivity had a mean AUC of 0.685 ± 0.069. A low level of structural connectivity between the left area 6 medial-anterior (6ma) and the right area superior temporal sulcus dorsal anterior (STSda) had the greatest impact on this model's output, while the rest of the features demonstrated a mix of low and high levels of connectivity, with great overlap (Figure 7C). At the network level, the language system had the greatest contribution to the model, followed by the SN, SMN, multiple demand network, and the VAN (Figures 7D, F).

#### 3.3.3. Functional ambulation category

**Figure 8:**
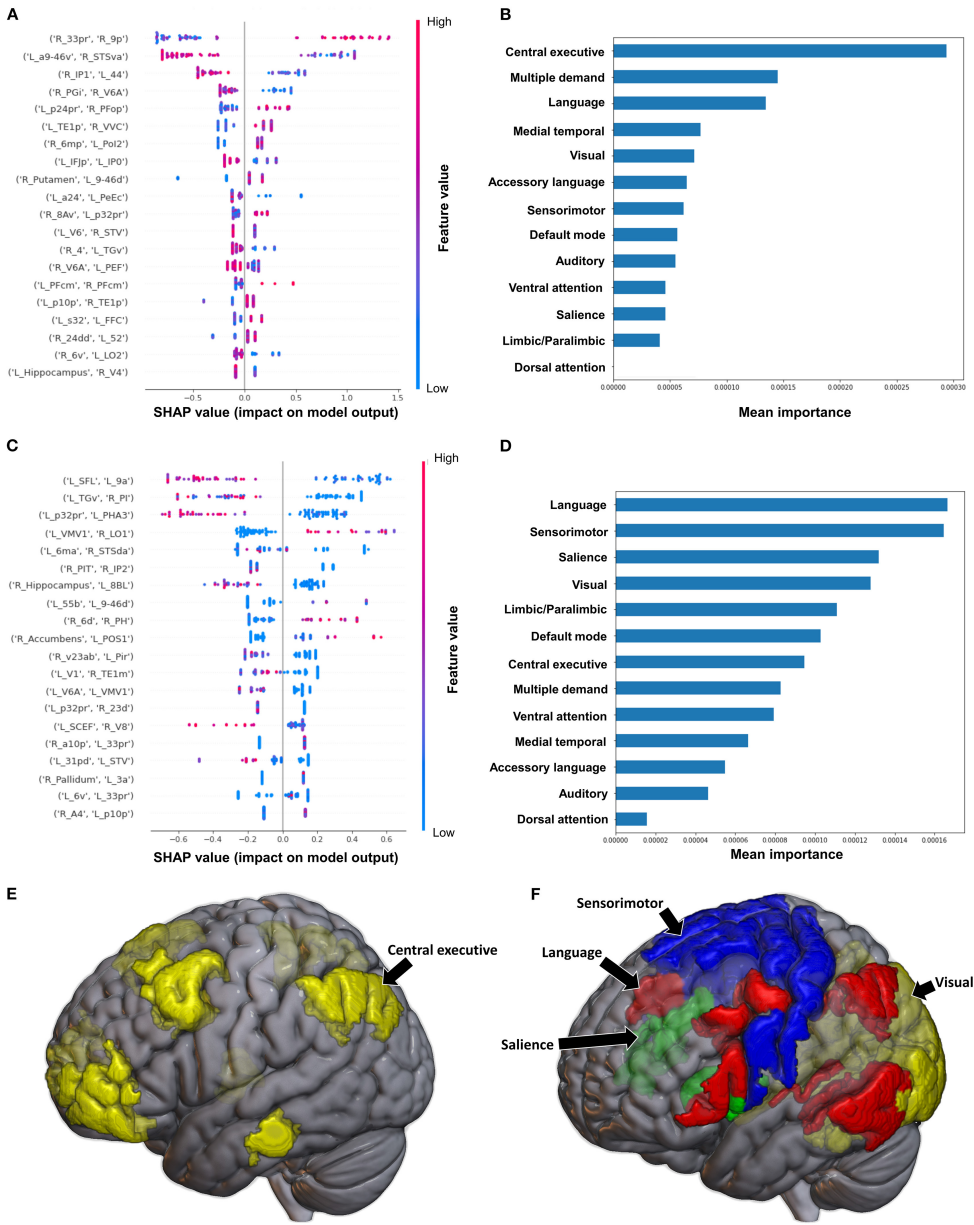
Feature Importance Analysis of the models classifying the functional ambulation category based on functional connectivity at the **(A)** individual brain region level, and **(B)** the network level; and based on structural connectivity at the **(C)** the individual brain region level, and **(D)** the network level. The networks contributing most to the models' classifications, based on **(E)** functional connectivity, and **(F)** structural connectivity, have also been visualized on brain models.

The model utilizing FC to predict FAC achieved a mean AUC of 0.75 ± 0.10 following hyperparameter tuning. At the individual brain region level, a high FC between the right area 33 prime (33pr) and right area 9 posterior (9p), and a low FC between the left area anterior 9-46v (a9-46v) and right STSva were the top two features contributing to the model (Figure 8A). The CEN demonstrated the greatest contribution to the model at the network level (Figures 8B, E).

When utilizing FC, the model achieved a mean AUC of 0.804 ± 0.115. A low connectivity between the left superior frontal language area (SFL) and left area 9 anterior (9a), left area temporal gyrus ventral (TGv) and right PI, and left p32pr and left parahippocampal area 3 (PHA3) were the top three features contributing to the model's output, though there was overlap among the top 20 features' contributions (Figure 8C). At the network level, the language system and the SMN demonstrated the greatest contribution, followed by the SN and the VN (Figures 8D, F).

## 4. Discussion

In this study, we utilized machine learning models to predict functional impairment after stroke using functional and structural connectivity and analyzed important features to identify the neuroanatomical basis of this impairment. Notably, our models achieved a high performance when classifying standardized tests of functional impairment. Our data also revealed broad disruptions across multiple brain networks in functional and structural connectivity, especially within the DAN and VAN. Together, our findings demonstrate the utility of our methodology to explore and discover the connectomic disturbances underlying stroke, and how networks respond to insults. While our study was the first comprehensive analysis utilizing both functional and structural connectivity to classify standardized tests of functional impairment using machine learning, the generalizability of our findings is limited by a relatively small and heterogeneous sample. The following discussion will therefore examine whether our findings may be explained by the existing literature, though we acknowledge that our hypotheses are speculative in nature and further studies are necessary to validate these claims.

### 4.1. Attention and language networks in stroke

Although our models pointed to several large-scale networks which are known to be important in functional recovery after stroke, such as the DMN, CEN, and SMN (Tuladhar et al., 2013; Larivière et al., 2018; Wu et al., 2020; Olafson et al., 2021; Vicentini et al., 2021), the attention networks, the DAN and VAN were among the top contributors in several of our models. Classically, The DAN is considered to be crucial for sustaining attention on a given task (Ptak and Schnider, 2010), while the VAN is thought to act as a circuit breaker by redirecting attention in response to salient events (Corbetta and Shulman, 2002; Corbetta et al., 2008). Generally, impairment in the DAN following stroke has been associated with neglect (Corbetta and Shulman, 2011; Barrett et al., 2019) and the severity of motor impairment (Siegel et al., 2016), however, the exact nature of changes within the attention networks in stroke patients remains unknown. Some studies have reported increased connectivity (Rehme et al., 2012; Wang et al., 2014; Siegel et al., 2016; Lee et al., 2018), contextualized by the greater need for patients to direct attention to movements following stroke. While others have demonstrated lower FC within the VAN and DAN in both task-free and task-based settings (He et al., 2007; Baldassarre et al., 2016; Adhikari et al., 2017; Barrett et al., 2019). This may be pointing to increased inter-network connectivity and decreased intra-network connectivity, representing the loss of network segregation and specialization following stroke (Caliandro et al., 2017; Marebwa et al., 2017; Guo et al., 2019). Interestingly, congruent to our findings, Romeo et al. recently demonstrated a positive correlation between decreased connectivity of the DAN and the motor component of the Functional Independence Measure (FIM) in the sub-acute to chronic period following stroke (Romeo et al., 2021). Furthermore, several studies have shown that the outcome of motor rehabilitation may be associated with preserved FC within the ipsilesional DAN (Cheng et al., 2021; D'Imperio et al., 2021). Therefore, patients with preserved connectivity within the DAN and VAN may be performing better across the tests in our models. We however cannot validate this given the task-free nature of our study. Nonetheless, it is evident that the attention networks play a role in mediating functional performance, though further studies on large, homogeneous samples are necessary to elucidate the mechanism behind this, which may provide further therapeutic avenues.

Interestingly, the language and accessory language networks were highlighted in our models classifying the BRS, FMA, Barthel Index, Semans balance scale and FAC. This may be due to impairments in the language system typically accompanying strokes with worse motor and functional impairment, and indeed those with expressive aphasia tend to have an 80% chance of also suffering hemiplegia (Romeo et al., 2021). Due to our small sample size, we were unable to perform separate analyses on patients reporting a brain lesion in the left compared to the right hemisphere. However, given most of the strokes within our cohort were subcortical, only nine out of 17 strokes affecting speech were left-sided cortical strokes, and most patients with motor deficits did not have speech deficits, we speculate that there may be alternative reasons for why the language network contributed to our classifications. This finding may instead be pointing to the functional symbiosis between language and motor function (recently reviewed by Anderlini et al., 2019). Recently, several studies have demonstrated that not only do deficits in these domains co-occur, but they also interact in their treatment. For example, Romeo et al. also demonstrated an association between the FIM motor score and the language network, specifically a higher integration within the region of the precentral gyrus, pointing to a possible role of the language network in motor sequence planning (Romeo et al., 2021). Indeed, Maitra et al. have reported an improvement in motor function through self-vocalization (Maitra et al., 2006), while Arya et al. demonstrated inadvertent improvement in language following upper limb therapy (Arya and Pandian, 2014). A similar effect was also found following transcranial direct current stimulation over the motor cortex, which led to improved language outcomes (Meinzer et al., 2016). More recently, Hybbinette et al. also demonstrated that similar mechanism of recovery may be involved in language and motor recovery post-stroke, further contributing to the so-termed “shared recovery hypothesis” (Hybbinette et al., 2021). While well-controlled studies examining this phenomenon are lacking, our findings may suggest either: functional compensation by the language system associated with better performance, or impaired connectivity within the language system manifesting in poor sensorimotor outcomes. Since our models are unable to point to which is the case, further studies are necessary to elucidate the interaction of the language and motor networks following stroke. Nonetheless, both our findings, and the literature highlight the possible need to simultaneously target the language and motor systems for effective neurorehabilitation.

### 4.2. The role of the limbic system in motor recovery

Our models classifying the BRS Upper Limb and FMA Upper Limb both suggested that the limbic/paralimbic system may be playing an important role in motor function following stroke. A similar finding was reported recently by Li et al., who found that the connectivity between the limbic network and DAN was predictive of the FMA (Nishimura et al., 2011). It is therefore possible that the limbic system may be contributing to motor learning after stroke. The limbic system in the Infinitome atlas comprises the orbitofrontal cortex, amygdala, hippocampus, along with other medial temporal regions. Previous studies have established that motor sequence learning relies on the interaction between the prefrontal cortex and hippocampal and striatal networks, along with cortico-cerebellar networks (Albouy et al., 2008; Fernández-Seara et al., 2009; Rose et al., 2011; Burman, 2019; Schapiro et al., 2019; Gann et al., 2021). The activity within the fronto-hippocampal networks decrease as learning progresses (Albouy et al., 2013). As motor learning and re-learning is a key part of neurorehabilitation in the post-stroke period, the limbic system may be key to this process. Alternatively, as the limbic system is also associated with reward and motivation, this finding may be implicating motivation as a key factor in performance. Although one study has examined the role of the motivation in motor recovery following spinal-cord injury in macaques, demonstrating increased functional connectivity between the primary motor cortex and the ventral striatum, orbitofrontal cortex, and anterior cingulate (Nishimura et al., 2011), the role of the limbic network in motor recovery in humans has not garnered much attention. However, it is widely assumed that motivation is a significant component of neurorehabilitation (Widmer et al., 2017; Oyake et al., 2020). Consequently, the limbic system may potentially be a target to improve motor recovery in the post-stroke period, though this remains conjectural and further studies are required to substantiate it.

### 4.3. Machine learning prediction of stroke outcomes

The prediction performance of our models was comparable to those reported in previous studies examining acute/subacute stroke patients. Wang et al. achieved an AUC of 0.899 using random forest models in acute stroke patients (Wang et al., 2019), while Thakkar et al. had an AUC of 0.77 (Thakkar et al., 2020). It should be noted that direct comparisons of performance are not possible, as most other studies have not combined both DWI based structural and rsfMRI-based FC metrics in machine learning models. Riahi et al. utilized EEG-based FC to estimate the FMA scores in chronic stroke, achieving an *R*^2^ of 0.97 in their regression model (Riahi et al., 2020). Tozlu et al. compared the performance of several types of models in predicting FMA scores based on a combination of demographic, clinical, neurophysiological, and structural connectivity based metrics, achieving an *R*^2^ between 0.70 and 0.91 (Tozlu et al., 2020). In order to compare and generalize the performance of our models, future research should incorporate functional and structural connectivity measures into holistic brain connectivity models.

Interestingly, our models utilizing FC tended to have better performance than those utilizing structural connectivity. While this needs to be further investigated and validated in independent cohorts, we speculate that this may be reflective of the rapidity of functional compensation, compared to the structural changes which may occur over a longer time span. This is in line with several studies suggesting that functional reorganization is observed within the first 4–5 weeks following stroke (Golestani et al., 2013; Nijboer et al., 2017; Xia et al., 2021), while structural changes occur from 3 to 12 months (Lin L. Y. et al., 2018); though these timings are contentious. For example, a recent study found no changes in the connectivity of motor areas over a year, despite motor improvement (Branscheidt et al., 2022). This may suggest that functional connectivity changes occur distributively to promote recovery, or that some reported connectivity changes are lesion-related, and not a form of compensation. These discrepancies must be addressed through much larger studies utilizing longitudinal data looking at whole-brain functional connectivity. Though there are a number of outstanding questions, our findings make clear that structural and functional connectome changes are related to functional outcomes, and that machine learning models may be necessary to elucidate the complex patterns of neural change occurring in response to stroke.

### 4.4. Future directions

Overall, our analysis highlighted several networks which may be playing key roles in motor recovery post-stroke, including the DAN, VAN, the language network, and the limbic system. It is possible that enhancing these networks through non-invasive stimulation techniques, such as repetitive transcranial magnetic stimulation, may improve functional outcomes post-stroke. This is however entirely speculative, and clinical trials are necessary to test these targets. rTMS has already demonstrated benefit for motor recovery the post-acute period, however, treatment often targets the primary motor cortex (Dionísio et al., 2018; Fisicaro et al., 2019). It is therefore necessary to investigate whether a more individualized network-based approach to target selection would improve outcomes. While some of our group has demonstrated that this may be a promising and efficacious technique in a case study (Yeung et al., 2021), larger, controlled trials are needed.

Our study also reveals the need to adopt a global-approach when examining deficits in stroke, and therefore looking beyond classically defined motor-regions when investigating motor deficits. This may consequently allow for the development of deficit-specific brain network—networks of functionally connected regions which may be associated with upper limb or lower limb function, or even a spasticity network. These may then enable for further identification of specific targets for treatment.

### 4.5. Limitations

Our study is limited by a small, heterogeneous sample, and our findings require larger scale prospective studies for validation. Owing to this, we cannot assume that our results are not biased by overfitting to our sample and class imbalances, though our methodology employs several approaches to mitigate this risk, including 5-fold cross-validation and early stopping criteria. Furthermore, future studies should attempt to replicate functional and structural connectivity-based models in a longitudinal cohort study to investigate whether connectivity changes may reveal pathological and compensatory mechanisms in the acute and chronic stages. Finally, models may further benefit from multimodal inputs, considering baseline information beyond connectomic data as we have done. This may enable the establishment of more individualized diagnostic trajectories for patients.

## 5. Conclusion

The brain connectivity changes that occur as a consequence of stroke are still poorly understood. Analyses of structural and functional changes associated with behavioral outcomes may begin to disentangle the deleterious effects of injury and the facilitatory effects of compensation and recovery; however, explainable machine learning methods are necessary to model and decode the complexity of brain response. Our study highlights the potential of machine learning methods combined with connectivity analysis in predicting outcomes in neurorehabilitation and disentangling the neural correlates of functional impairments, though further longitudinal studies are necessary.

## Data availability statement

The raw data supporting the conclusions of this article will be made available by the authors, without undue reservation.

## Ethics statement

The studies involving human participants were reviewed and approved by the Ethical Committee of the Affiliated Brain Hospital of Nanjing Medical University. The patients/participants provided their written informed consent to participate in this study.

## Author contributions

RB conceived and designed the study. LL and WL performed the study and collect materials. NM wrote the code. NM and KO analyzed the results. OT and KO visualized the results. OT, KO, and XH wrote the manuscript. IY, XZ, MS, SD, RB, MH, and LL helped coordinate the study and reviewed the manuscript. All authors contributed to the article and approved the submitted version.
